# Development of a sustainable multianalyte MEKC method for quantitation of the antihyperlipidemic drugs ezetimibe together with three statins. Greenness and whiteness appraisal studies

**DOI:** 10.1186/s13065-023-01040-y

**Published:** 2023-09-23

**Authors:** Haydi S. Elbordiny, Sohila M. Elonsy, Hoda G. Daabees, Tarek S. Belal

**Affiliations:** 1 Pharmaceutical Chemistry Department, Faculty of Pharmacy, Damanhour University, Damanhour, Egypt; 2 Pharmaceutical Analytical Chemistry Department, Faculty of Pharmacy, Damanhour University, Damanhour, Egypt; 3 Pharmaceutical Analytical Chemistry Department, Faculty of Pharmacy, University of Alexandria, Elmessalah, Alexandria, 21521 Egypt

**Keywords:** Ezetimibe, Atorvastatin, Rosuvastatin, Simvastatin, MEKC, Whiteness and greenness evaluation

## Abstract

Implementing powerful and sustainable research that complies with green analytical chemistry (GAC) and white analytical chemistry (WAC) fundamentals can downsize the environmental compliance costs and fruitfully affects practical and economic issues. Within this framework, rapid and white analytical micellar electrokinetic capillary chromatography (MEKC) methodology was developed for the synchronized estimation of the antihyperlipidemic drugs Ezetimibe (EZE), Atorvastatin (ATO), Rosuvastatin (ROS) and Simvastatin (SIM). The technique was established using fused silica capillary (50 cm, 50 µm id) and the background electrolyte was 0.025 M borate buffer pH 9.2 containing 0.025 M sodium dodecyl sulfate (SDS) and 10% v/v acetonitrile as the organic modifier. Diode array detector was adjusted at 243 nm for ATO and ROS and 237 nm for EZE and SIM. Separation was accomplished within 10 min with migration times of 4.12, 5.42, 8.23 and 8.74 min for ROS, ATO, EZE and SIM respectively. The 4 drugs were quantitated in the concentration range of 10–100 μg/mL and the correlation coefficients were not less than 0.9993. The high sensitivity was illustrated by values of the detection and quantitation limits. The limits of detection for ROS, ATO, EZE and SIM were 0.52, 0.75, 0.42 and 0.64 μg/mL, respectively, whereas, the limits of quantitation values were 1.73, 2.50, 1.40 and 2.13 μg/mL for the studied drugs, respectively. In addition to validation, as reported by the ICH guidelines, greenness and whiteness assessment using the novel AGREE calculator and the holistic functionality model RGB12 were performed. The results proved the efficiency and whiteness of the suggested technique to be routinely implemented in quality control laboratories for the assay of the four drugs and the binary mixtures of EZE with either ATO, ROS or SIM in fixed-dose combined tablets.

**Supplementary Information:**

The online version contains supplementary material available at 10.1186/s13065-023-01040-y.

## Introduction

“What you don’t have, can’t leak” as Trevor Kletz [1] declared in 1978 and described the concept of “Inherent safety” which infers that preventing is the key approach rather than controlling the resulting hazards. This initiative work was the cornerstone for the evaluation of green chemistry followed by GAC concepts at the dawn of the twenty-first century. In addition to the newfangled approach of WAC that has been established as an amalgamation of the GAC and the perception of sustainable development. In 2013, Gałuszka et al. [2] proposed the 12 GAC principles whereas Nowak et al*.* outlined the 12 WAC assumptions in 2021 [3]. A comprehensive study of GAC and WAC principles revealed the harmony between principles 2,7,8,9 in GAC and 1,6,7,9,10 and 11 in WAC. These principles explained the strategy of resolving complex multianalyte samples using power-saving techniques such as capillary electrophoresis, consuming minute volumes of solvents and producing minimum waste in one single short run. In our endeavor to stick to the GAC and WAC protocols, this strategy is fully implemented by our research group and is powerfully endorsed for application in quality control units and the research arena.

**Fig. 1:**
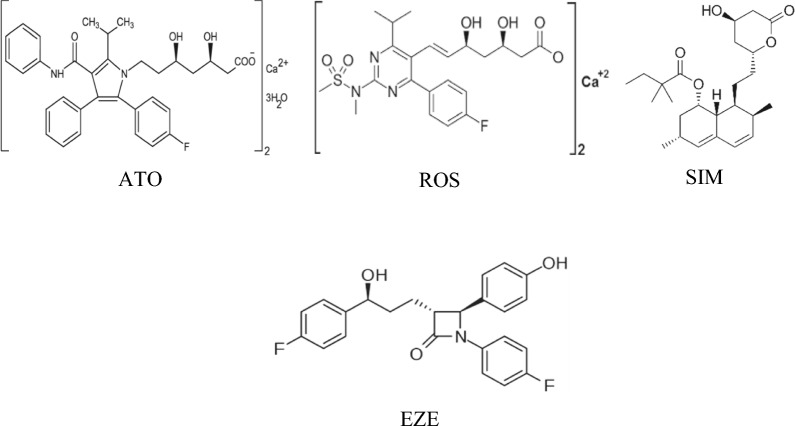
Chemical structures of the antihyperlipidemic drugs Atorvastatin (ATO), Rosuvastatin (ROS), Simvastatin (SIM) and Ezetimibe (EZE)

Statins are well known for their hydroxy-methyl-glutyryl-CoA (HMG-CoA) reductase inhibition and their pivotal action on cholesterol biosynthesis. Consequently, they are considered the most extensively used antihyperlipidemic indicated for the management and prevention of cardiovascular and coronary heart diseases. Nowadays, a great concern emerged to pinpoint numerous off-target effects of statins. These cholesterol-independent actions include anti-inflammatory, anti-thrombotic, anticancer, and neuroprotection in addition to immunomodulatory functions [4–11]. Over the past 2 years, drug repurposing and many pharmacotherapies investigations were the scientists' and researchers' priorities, to confront the pandemic COVID-19. In this context, in silico studies illustrated the usefulness of statins in the treatment of COVID-19 by these suggested mechanisms either by inhibiting SARS-CoV-2 main protease [12] or SARS-CoV-2-RNA dependent RNA polymerase [13]. On the other hand, ezetimibe (EZE) had an add-on effect to the ongoing statin therapy due to the inhibition of cholesterol absorption at the jejunal enterocyte brush border. For patients who did not achieve normal cholesterolemia using statin monotherapy, the combination of statins and EZE in a single pill will serve as a golden alternative [14]. Figure 1 shows the chemical structures of the 4 studied antihyperlipidemic drugs.

Surveying the literature revealed that different mixtures of EZE with either ATO, ROS or SIM were assayed using various techniques: spectrophotometry [15–22], spectrofluorimetry [23, 24], HPLC [19, 25–35], HPTLC [34–37], UPLC [38], MEKC [39, 40] and CE [41]. However, there is no reported capillary electrophoresis (CE)-based method for the synchronized determination of the 3 statins together with EZE. The development of multi-analyte, broad-spectrum analytical platforms for the separation and synchronized quantitation of several structurally or pharmacologically related drugs [42–46] is one of the advantageous analytical trends regarding GAC principles [2]. Such multi-analyte, multiuse methods (principle 8 of GAC) are time and energy-saving and thus more economic (principle 9), they also require fewer samples (principle 2) and therefore reduce the generated waste (principle 7). Accordingly, such analytical platforms are valuable during routine analysis in quality control laboratories as they could be applied for the assay of different pharmaceutical samples.

The ultimate goal of the study was to establish a white method for the fast determination of the most prescribed antihyperlipidemic drugs together during a single run using the green technique MEKC, thereby reducing cost, time, and solvent used, saving the environment and enlarging the scope of application of the method to include the four drugs and analyze different marketed tablet dosage forms.

## Experimental

### Materials

ATO, SIM and EZE were kindly gifted from Pharco Pharmaceuticals Co., Alexandria, Egypt while ROS was obtained from Borg Pharmaceutical Industries, Alexandria, Egypt. The tablet dosage forms were purchased from local Pharmacies and they include Atoreza^®^ (Marcyrl Pharmaceutical Industries, Egypt) labelled to contain 10 mg ATO and 10 mg EZE per tablet, Cholerose plus^®^ (Marcyrl Pharmaceutical Industries, Egypt) labelled to contain 10 mg ROS and 10 mg EZE per tablet, and Simv-Eze^®^ (Pharco Pharmaceuticals Co., Egypt) labelled to contain 10 mg SIM and 10 mg EZE per tablet. Analytical-grade boric acid, sodium hydroxide and sodium dodecyl sulphate (SDS) were purchased from El-Nasr Chemical Company (Egypt). HPLC-grade methanol and acetonitrile were obtained from Sigma-Aldrich (Germany).

### Instrumentation and conditions of MEKC separation

Agilent CE instrument 7100 series (Agilent Technologies Deutschland, GmbH, Waldbronn, Germany) was used and it is equipped with a Diode Array Detector and a data handling system comprising a computer loaded with Agilent ChemStation Software. The optimized method applied a deactivated fused silica capillary (Agilent Technologies, Waldbronn, Germany) with the following specifications: 58.5 cm full length, 50 cm effective length, and 50 μm internal diameter. The Diode array detector was adjusted at 243 nm for ATO and ROS, and 237 nm for EZE and SIM.

At the commencement of each working day, the capillary was cleaned with 0.5 M NaOH for 15 min then with water for 15 min. After that, it was washed with 0.1 M NaOH for 300 s, waiting for 150 s to reach through activation of the internal capillary wall, washed with water for 300 s and then conditioned with the running buffer for 600 s. The capillary was cleaned with a running buffer for a couple of minutes between each two consecutive injections. Buffer vials were refilled after each 5 successive runs to sustain appropriate reproducibility among consecutive injections. Injections were done under hydrodynamic mode using a pressure of 50 mbar for 10 s. The adjusted voltage was 30 kV.

The optimum working buffer was borate buffer (0.025 M, pH 9.2) which was prepared by weighing 309 mg of boric acid, and 100 mg of sodium hydroxide in 100 mL of distilled water, then pH was checked and adjusted. To prepare 0.025 M of SDS in buffer, 722 mg of SDS was added to 100 mL of the formerly made buffer and then sonicated for 10 min till thorough solubilization of SDS powder. The final working background electrolyte (BGE) comprised 90 parts of 0.025 M borate buffer (pH 9.2) containing 0.025 M SDS and 10 parts of HPLC-grade acetonitrile.

### Preparation of stock and working solutions and plotting of the calibration graphs

Standard stock solutions containing 1000 μg/mL of EZE, ATO, ROS and SIM were independently prepared in HPLC-grade methanol. Working solutions were made by relocating appropriate volumes of the stock solutions into a series of 10 mL calibrated flasks to attain the common concentration range of 10–100 μg/mL. Finally, flasks were adjusted to 3 mL methanol and then completed to the mark with distilled water to avoid precipitation of EZE and SIM. Three injections were done for each solution. Peak areas were plotted as a function of the corresponding concentrations to establish the calibration graphs.

### Preparation of sample solutions. assay of atoreza^®^, cholerose plus^®^ and Simv-Eze^®^ tablets

Seven tablets of each of the abovementioned brands were weighed and thoroughly crushed. For each assayed formulation, a volume of 15 mL methanol was added to an accurate mass of the mixed powder equivalent to 25 mg EZE and 25 mg of either ATO or ROS or SIM. Each solution was sonicated for 15 min and then filtered into a 25 mL calibrated flask. The residue was washed with 2 × 3 mL methanol and washes were added to the filtrate which was completed to final volume with methanol. Accurately measured volumes of the filtered tablet solutions were relocated into a series of 10 mL calibrated flasks to get the concentration range 10–100 μg/mL. Sample solutions were adjusted to 3 mL using methanol, diluted to volume with distilled water and finally analyzed as per formerly cited. The recovery values were computed from the analogously analyzed external standards.

For standard addition analysis, accurate volumes of EZE, ATO, ROS and SIM standard solutions were added to sample solutions to obtain aggregate concentrations inside the specified range and then analyzed as formerly detailed. Recovery values were calculated by relating the analyte response with the increased response measured after the addition of the standard.

## Results and discussion

### Evolution of the MEKC method

The separation of the four antihyperlipidemic drugs was challenging owing to their different physicochemical properties. The encountered problems were the solubility of EZE and SIM in addition to their peak overlap. Preliminarily trials were performed using capillary zone electrophoresis (CZE) mode with 0.05 M of either acetate buffer pH 4.7, phosphate buffer pH 7.4 or borate buffer pH 9.2, unfortunately, these trials resulted in EZE and SIM precipitation in all these conditions. On the other hand, ATO and ROS were efficiently resolved from each other with suitable peak shapes and migration times.

SIM had a pKa value of 14.91 with very poor water solubility (0.0122 mg/mL) [47]. As a result, it does not ionize in the conventional pH range (2.0–12.0) of CZE. Consequently, MEKC will be the method of choice for the estimation of SIM. MEKC is a hybrid technique that amalgamates chromatographic and electrophoretic methods of analysis. The chief concept involves the addition of surfactant mainly SDS beyond its critical micelle concentration (CMC) to the buffer solution. It had many pros in the separation of neutral drugs not only the ionic drugs as in case of conventional CZE, in addition to solubilization of the poorly soluble drugs like EZE and SIM in our case. Furthermore, the improvement of both the resolution and peak shapes of the cited drugs is quite noticed. In this mode, the electro-osmotic flow (EOF) simulates the action of the mobile phase in traditional chromatography [48, 49].

Logically, the anionic surfactant will be directed towards the anode by electrostatic attraction while the EOF pushes the entire solution to the cathode due to the negatively charged silica on the inside surface of capillaries. However, practically, micelles move towards the cathode too but with slower velocity than the EOF, as the EOF is predominant over the micelle’s electrophoretic mobility. Generally, partitioning of the solute between micelle and bulk solution is the key determinant of the migration time. The higher the affinity of the analyte to micelles, the longer the migration time and vice versa [49]. The aforementioned reasons explain the migration order of anionic, cationic and neutral compounds. According to the charge on the analyte, electrostatic repulsion exists between anions and SDS micelles due to the similarity of negative charge on both, so anionic samples move with the bulk solution and their migration times are nearby the EOF. On the contrary, cations are strongly attracted to the micelle, so they are eluted at close proximity to the slow micelle electrophoretic mobility. Additionally, the hydrophobicity of the analytes influences their elution order. It was noticed that hydrophobic compounds will be incorporated into the surfactant micelles, so they are eluted with the slower micelles’ velocity. While hydrophilic analytes will elute first with the EOF. It is necessary to mention that the neutral compounds depend on the hydrophobicity basis of separation [49]. The last approach in unresponsive drug separation was to introduce an organic solvent either methanol or ACN to the buffer system, as the organic modifier affects the EOF mainly by altering the zeta potential and the buffer viscosity [50]. In this study, ACN gave excellent results in terms of peak shapes, resolution and migration times.

The study of various experimental settings included buffer pH and concentration, surfactant concentration, organic modifier, dilution solvent, voltage, selection of injection time and finally the measuring wavelength.

#### Buffer pH

The role of pH of the buffer is crucial relative to the extent of ionization of drugs. Different buffers were examined: phosphate buffer pH 7.4 and borate buffers (pH 7–10) in the presence of 0.02–0.025 M SDS. The separation order was unaffected: ROS, ATO, EZE and SIM. However, in most experiments, all peaks required longer migration times, and EZE and SIM had distorted peak shapes as presented in Additional file 1: Figs. S1, S2 in the Additional file. Finally, it was observed that 0.025 M borate buffer (pH 9.2) and 0.025 M SDS produced better peak shapes and resolution with suitable and reproducible migration times.

#### Buffer concentration

Buffer concentration had a great contribution to CE-based separation methods. The outcome of buffer concentration was tested by using borate buffer with concentrations 0.01, 0.025 and 0.05 M at pH 9.2. Results revealed that at lower buffer concentration (0.01 M), peaks of EZE and SIM were separated with insufficient resolution (about 1.24), while 0.05 M borate showed distorted EZE and SIM peaks with longer migration times (11.1 and 12.31 min respectively) as illustrated in Additional file 1: Figure S1 and Table S1 in the Additional file. Finally, 0.025 M concentration of borate buffer was of choice to get the best electrophoretic separation in an acceptable run time.

#### Effect of dilution solvent

As long as CE is deemed as a green technique, water is the main diluent used. However, in our study, the volume of working solutions was adjusted to 3 mL methanol and then completed to volume with water. This quantity of methanol was the least amount that could be used to ensure the solubilization of SIM and EZE while maintaining the suitable peak shape and resolution.

#### Adjusted voltage

The variation of the applied voltage (15, 20, 25 and 30 kV) was studied using the developed BGE. As expected, it was perceived that by reducing voltage, migration velocity declines due to a decrease of EOF. In all voltages, the resolution was unchanged, however, shorter migration times and therefore briefer total run time was reached upon using 30 kV which was assigned as the optimal voltage.

#### Surfactant concentration

The outcome of SDS concentration on mixture separation was studied by the addition of 0.020 and 0.025 M SDS in the BGE in 50 cm capillary. SDS (0.025 M) showed excellent separation profile and migration times.

#### Organic modifier

The type and amount of organic modifier have a great impact on the separation pattern of the tested drugs. EZE and SIM peaks were broad and shifted to longer migration time upon using 10% (v/v) methanol as demonstrated in Additional file 1: Figure S2 in the Additional file. Upon using 15–25% (v/v), erroneous results were noticed, as the high content of the organic modifier can hinder micelle formation from SDS monomers. It is generally believed that micelles are not stable in mixtures of water and organic solvents containing more than 20–30% of organic solvents [51]. Favourably, using acetonitrile instead of methanol provided excellent electrophoretic separation with reasonable migration times for the four candidates. Therefore 10% (v/v) of acetonitrile was selected.

#### Injection time

In hydrodynamic injection, injection time is directly correlated to peak height and width. To investigate the effect of injection time, sample solutions were injected at 50 mbar with different times from 2 to 14 s. As injection time increases, peak height rises relatively; however, more increase in injection time causes peak shape deformity. The best injection time was 10 s because it produced acceptable symmetric peaks.

#### Measuring wavelengths

DAD has the advantage of generating separation electropherograms at different wavelengths for the same run. Therefore, each drug could be accurately measured at its maximum absorption wavelength, thus boosting sensitivity of the procedure. In addition, DAD acts as a peak purity inspector. Additional file 1: Figures S3, S4, S5, S6 in the Additional file show the UV spectra of the studied drugs in the selected BGE. ROS and ATO were measured at 243 nm while EZE and SIM were quantified at 237 nm.

The ionization constants and chemical structures of the four analytes together with the presence of SDS micelles clarified the elution behavior for this quaternary mixture. The aforementioned arguments explained the migration order in MEKC generally: anions, neutral analytes and finally cations [49]. The pKa values of ROS, ATO, EZE and SIM are 4.0, 4.33, 9.48 and 14.91 respectively [47, 52–54]. Both ROS and ATO exist as carboxylic acid salts. At pH 9.2, the carboxylate salts ROS (pKa 4.0) and ATO (pKa 4.33) will be negatively charged. In contrast, EZE (pKa 9.48) is almost neutral while SIM (pKa 14.91) most probably will be positively charged. The anionic compounds ROS and ATO are expected to move freely and elute faster, while EZE and SIM will be incorporated into the micelles which will delay their migration. Furthermore, the higher molecular weight of ATO and its structural bulkiness obviously explain its elution after ROS although they have quite similar pKa values. Similar elution patterns were reported in MEKC separations of ATO-EZE [40] and EZE-SIM [50] binary mixtures.

**Fig. 2:**
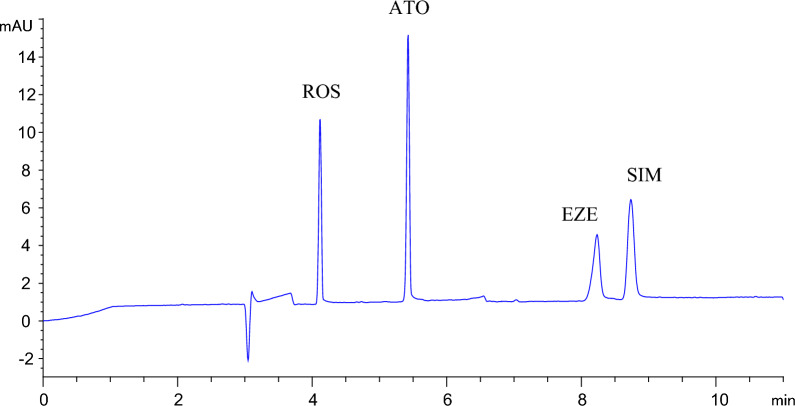
MEKC electropherogram of a standard mixture containing 70 µg/mL ROS, 70 µg/mL ATO, 30 µg/mL EZE and 30 µg/mL SIM at 237 nm

The described method enabled separation of the four antihyperlipidemic drugs within 10 min as shown in the MEKC electropherogram in Fig. 2. Recorded migration times were 4.12, 5.42, 8.23 and 8.74 min for ROS, ATO, EZE and SIM respectively. The developed method revealed adequate resolution values not less than 2.64, along with other system suitability parameters which were found reasonable (Additional file 1: Table S2 in the Additional file).

### Method validation

The described method was validated in agreement with the International Conference on Harmonization (ICH) guidelines [55].

#### Linearity and concentration ranges

The linear relationship between integrated peak areas and the corresponding concentrations of the four analytes within the cited range (10–100 μg/mL) was evaluated using regression analysis. The values of correlation coefficients were nearly equal to unity, and acceptable statistical linearity parameters calculated and recorded in Table 1 proved the good linearity.

**Table 1:** Statistical analytical parameters for determination of ROS, ATO, EZE and SIM mixture using the proposed MEKC-DAD method

Parameter	ROS	ATO	EZE	SIM
Wavelength (nm)	243	243	237	237
Concentration range (μg/mL)	10–100	10–100	10–100	10–100
Intercept (a)	− 0.493	− 0.393	− 7.167	4.104
S_a_^a^	0.283	0.520	1.224	0.668
Slope (b)	0.506	1.210	1.565	0.768
S_b_^b^	0.005	0.008	0.020	0.009
RSD% of the slope (S_b_%)	0.99	0.66	1.28	1.17
Correlation coefficient (r)	0.9996	0.9998	0.9993	0.9995
S_y/x_^c^	0.414	0.762	1.792	1.037
F^d^	12,336	20,842	6290	7662
Significance F	4.83 × 10^‒14^	5.93 × 10^‒15^	7.12 × 10^‒13^	3.24 × 10^‒13^
LOD^e^ (μg/mL)	0.52	0.75	0.42	0.64
LOQ^f^ (μg/mL)	1.73	2.50	1.40	2.13

^a^Standard deviation of the intercept

^b^Standard deviation of the slope

^c^Standard deviation of residuals

^d^Variance ratio, equals the mean of squares due to regression divided by the mean of squares about regression (due to residuals)

^e^Limit of detection

^f^Limit of quantification

#### Detection and quantification limits

The values for detection and quantitation limits for the four drugs were calculated and elucidated in Table 1 according to the ICH guidelines. The LOD is defined as the concentration level that has a signal-to-noise ratio of 3:1, while for LOQ the ratio is considered to be 10:1. Obtained values validated the high sensitivity of the suggested MEKC-DAD platform.

#### Precision and accuracy

The within-day precision (Repeatability) and accuracy for the developed MEKC platform were appraised using three concentrations for each drug with three replicate analyses for each concentration on the same day. Likewise, the between-days precision (Intermediate Precision) and accuracy were inspected by analyzing the same three concentrations for each compound with three replicates on three days. Recoveries were computed using the regression equations and the results were satisfying (Table 2). The Percentage relative standard deviation (RSD %) and percentage relative error (Er %) did not exceed 2% (Table 2), supporting the high precision and accuracy of the optimized MEKC method for the assay of the investigated drugs.

**Table 2:** Precision and accuracy for determination of ROS, ATO, EZE and SIM using the proposed MEKC–DAD method

Analyte	Type of analysis	Nominal value (μg/ml)	Found ± SD^a^ (μg/ml)	RSD (%)^b^	E_r_ (%)^c^
ROS	Within-day	20	20.01 ± 0.08	0.40	0.05
		40	40.04 ± 0.19	0.48	0.10
		80	80.14 ± 0.62	0.77	0.18
	Between-days	20	19.86 ± 0.25	1.26	− 0.70
		40	39.82 ± 0.29	0.73	− 0.45
		80	79.94 ± 0.59	0.74	− 0.07
ATO	Within-day	20	20.14 ± 0.19	0.94	0.70
		40	40.13 ± 0.39	0.97	0.33
		80	80.04 ± 0.21	0.26	0.05
	Between-days	20	19.87 ± 0.31	1.56	− 0.65
		40	40.03 ± 0.44	1.10	0.08
		80	79.94 ± 0.35	0.44	− 0.07
EZE	Within-day	20	20.20 ± 0.11	0.55	1.00
		40	40.03 ± 0.11	0.28	0.08
		80	80.19 ± 0.33	0.41	0.24
	Between-days	20	20.13 ± 0.22	1.09	0.65
		40	39.82 ± 0.39	0.98	− 0.45
		80	80.15 ± 0.48	0.60	0.19
SIM	Within-day	20	20.02 ± 0.14	0.70	0.10
		40	40.37 ± 0.09	0.22	0.93
		80	80.02 ± 0.25	0.31	0.03
	Between-days	20	19.92 ± 0.38	1.91	− 0.40
		40	40.18 ± 0.34	0.85	0.45
		80	79.93 ± 0.34	0.43	− 0.09

^a^Mean ± standard deviation for three determinations

^b^% Relative standard deviation

^c^% Relative error

#### Selectivity

Method selectivity was assessed using various laboratory-prepared quaternary mixtures of the cited drugs with different concentration levels. The prepared mixtures were analysed, and generated results were gathered in Additional file 1: Table S3 in the Additional file. Found concentrations, RSD % and Er % were excellent which supported the suitability of the suggested MEKC platform for analysis of complex mixtures of the four antihyperlipidemic drugs.

#### Robustness

Testing the robustness of an analytical method is defined as a measurement of its aptitude to remain uninfluenced by small planned variations in experimental parameters. It indicates the method’s consistency during regular practice. Robustness of the optimized MEKC method was evaluated by calculating the SD and RSD of both peak areas and migration times after slight variations in the experimental settings. The considered parameters are borate buffer concentration 0.025 ± 0.002 M, buffer pH 9.2 ± 0.2, SDS concentration 0.025 ± 0.002 M, %v/v acetonitrile 10 ± 2% and wavelength ± 2 nm. The planned alterations did not intensely influence peak areas or migration times of the analysed drugs as evidenced by RSD% values that did not surpass 2% for both peak areas and migration times (Table 3), therefore, method robustness has been verified.

**Table 3:** Robustness evaluation for the analysis of ROS, ATO, SIM and EZE using the proposed MEKC-DAD method

Parameter	ROS			
	Peak area ± SD	RSD%	Migration time ± SD	RSD%
Buffer concentration (25 mM ± 2 mM)	20.93 ± 0.40	1.91	4.03 ± 0.02	0.50
Buffer pH (9.2 ± 0.2)	21.37 ± 0.40	1.87	4.08 ± 0.02	0.49
SDS concentration (25 mM ± 2 mM)	21.10 ± 0.36	1.71	4.04 ± 0.01	0.25
%v/v acetonitrile (10 ± 2%)	21.43 ± 0.42	1.96	4.02 ± 0.01	0.25
Wavelength (243 ± 2 nm)	21.20 ± 0.20	0.94		

Parameter	ATO			
	Peak area ± SD	RSD%	Migration time ± SD	RSD%
Buffer concentration (25 mM ± 2 mM)	60.90 ± 0.46	0.76	5.25 ± 0.02	0.38
Buffer pH (9.2 ± 0.2)	60.93 ± 0.40	0.66	5.33 ± 0.08	1.50
SDS concentration (25 mM ± 2 mM)	60.87 ± 0.40	0.66	5.37 ± 0.03	0.56
%v/v acetonitrile (10 ± 2%)	61.07 ± 0.70	1.15	5.37 ± 0.04	0.75
Wavelength (243 ± 2 nm)	61.00 ± 0.30	0.49		

Parameter	EZE			
	Peak area ± SD	RSD%	Migration time ± SD	RSD%
Buffer concentration (25 mM ± 2 mM)	60.90 ± 0.36	0.59	8.01 ± 0.09	1.12
Buffer pH (9.2 ± 0.2)	61.17 ± 0.35	0.57	8.09 ± 0.07	0.87
SDS concentration (25 mM ± 2 mM)	61.10 ± 0.20	0.33	8.08 ± 0.03	0.37
%v/v acetonitrile (10 ± 2%)	61.03 ± 0.35	0.57	8.00 ± 0.04	0.50
Wavelength (237 ± 2 nm)	61.20 ± 0.20	0.33		

Parameter	SIM			
	Peak area ± SD	RSD%	Migration time ± SD	RSD%
Buffer concentration (25 mM ± 2 mM)	63.12 ± 0.25	0.40	8.64 ± 0.07	0.81
Buffer pH (9.2 ± 0.2)	62.92 ± 0.19	0.30	8.63 ± 0.10	1.16
SDS concentration (25 mM ± 2 mM)	63.10 ± 0.36	0.57	8.61 ± 0.06	0.70
%v/v acetonitrile (10 ± 2%)	62.90 ± 0.46	0.73	8.65 ± 0.10	1.16
Wavelength (237 ± 2 nm)	63.18 ± 0.23	0.36		

Robustness parameters were determined for a mixture containing 60 µg/mL of ROS, ATO, SIM and EZE

#### Stability of solutions

The stock solutions of ATO, ROS, EZE and SIM were stable for at least one week at approximately 4 °C. The high recovery results, with %RSD < 2%, obtained from assaying these solutions proved the stability without major variations in measured responses or separation behavior generally.

### Analysis of tablets formulations

The developed MEKC platform was effectively utilized for the analysis of EZE and its binary mixtures with ATO, ROS and SIM in Atoreza^®^, Cholerose plus^®^ and Simv-Eze^®^ tablets. No interfering peaks were observed from any excipients or the dosage form matrix (Additional file 1: Figures S7, S8, S9 in the Additional file). Recovery values were appraised using both external standard and standard addition analyses. The assay outcomes exposed good accuracy and precision as believed from % recovery, SD, and RSD% values (Table 4). It is clear from these outcomes that the proposed platform is valid for the routine assay of EZE in its fixed-dose combinations with ATO or ROS or SIM with facile sample preparation and agreeable accuracy, precision and selectivity.

**Table 4:** Application of the proposed MEKC method to the analysis of ROS, ATO, and SIM in their binary dosage forms with EZE

Cholerose-plus^®^	External standard		Standard addition	
	ROS	EZE	ROS	EZE
%Recovery ± SD^a^	100.48 ± 0.68	99.37 ± 0.30	99.16 ± 0.74	100.18 ± 0.84
RSD%^b^	0.68	0.30	0.75	0.84

Atoreza^®^	External standard		Standard addition	
	ATO	EZE	ATO	EZE
%Recovery ± SD^a^	99.79 ± 0.78	100.74 ± 0.84	99.17 ± 0.73	99.51 ± 1.16
RSD%^b^	0.78	0.83	0.74	1.17

Simva-Eze^®^	External standard		Standard addition	
	SIM	EZE	SIM	EZE
%Recovery ± SD^a^	99.75 ± 0.35	99.57 ± 0.11	99.17 ± 0.30	100.14 ± 1.91
RSD%^b^	0.35	0.11	0.30	1.91

Quantification was carried out at 243 nm for ROS and ATO while at 237 nm for SIM and EZE

^a^Mean ± standard deviation for three determinations

^b^% Relative standard deviation

### Sustainability, greenness and whiteness evaluation protocols

Recently, it has been widely acceptable that accomplished analytical platforms should be assessed using at least one greenness assessment metric and/or multicriteria approach tools in order to ensure analytical performance, sustainability, benign environmental impact and economic cost. The trendiest metrics used were the Analytical GREEnness Metric Approach and Software (AGREE) [45, 56–59] and the RGB 12 model [3, 58, 59]. The proposed method was compared in the matter of greenness and whiteness with different selected reported techniques: spectrophotometry [22], HPLC–UV [60], gas chromatography with flame ionization detection (GC-FID) [60], ultra‐high‐performance supercritical fluid chromatography (UHPSFC) [61], HPTLC [62] and ultra-performance liquid chromatography-tandem triple quadrupole compound linear ion trap mass spectrometry (UPLC-Q-TRAP/MS) [63].

The AGREE method is the best and most automated facile tool that covers the 12 green analytical chemistry (GAC) fundamentals. The ideal green method had a score of 1 taking dark green color illustrated in a specific pictogram. Commonly, MEKC is greener than other spectrophotometric and chromatographic techniques including HPLC and HPTLC due to the minute amount of solvent used and nearly no waste produced. The pictograms displayed in Table 5 corroborated the top-ranking greenness of the optimized MEKC method with a score of 0.95 followed by GC-FID [60] (0.86), the spectrophotometric method [22] (0.77), HPTLC [62] (0.76), HPLC [60] and UHPSFC [61] (both 0.73) and finally UPLC-Q-TRAP/MS [63] (0.68). It is worth noticed that although UHPSFC and UPLC-Q-TRAP/MS are considered greener versions of HPLC due to low consumption of organic solvents and low waste production, however, they are highly consuming energy especially UPLC-Q-TRAP/MS due to mass detector in addition to their inaccessibility. Furthermore, UPLC-Q-TRAP/MS exhibited a multistep sample preparation procedure that elevated the penalization in the assessment process.

**Table 5:**
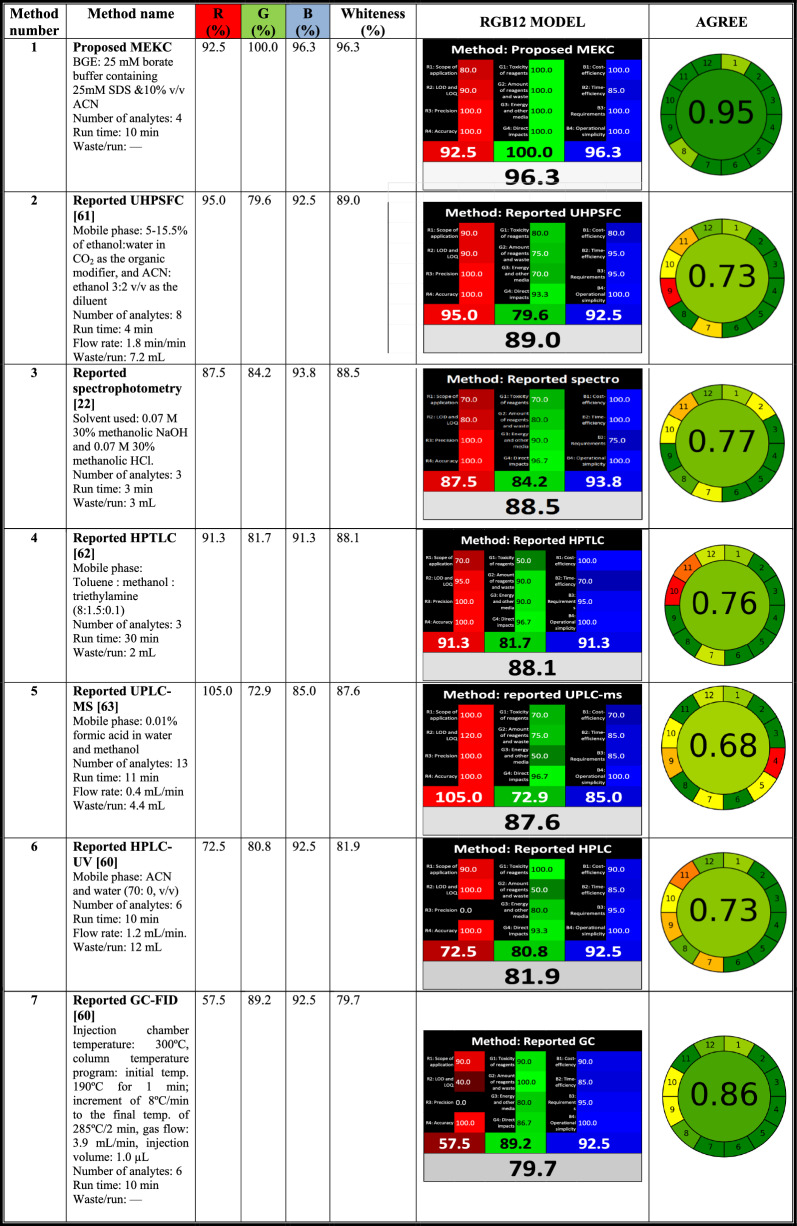
Evaluation of greenness (AGREE) and whiteness (RGB12 model) for the proposed MEKC and reported methods

Recently, Nowak et al. suggested a new version of the Red Green Blue (RGB) 12 design, which is divided into three areas, each area is expressed by a color and it contains specific parameters that assess important features of the analytical procedure [3]. Red areas evaluate analytical performance concerning validation principles. The green areas represent GAC principles and the blue areas describe productivity and practicability. Application of the sustainability concept and the novel whiteness approach in pharmaceutical analysis have been introduced by our research group [58, 59]. The suggested method was reviewed and compared to the relevant reported methods. Results of this comparison study are gathered in Table 5 and in the bar chart illustrated in Additional file 1: Figure S10 in the Additional file. The results of the investigation showed that:

Regarding validation criteria (red area), the largest scope of application is found in the reported UHPSFC [61] and HPLC [60] followed by the proposed MEKC method. All the methods are found accurate and precise since they followed the ICH recommendations, except the UHPSFC method [61] which adopted the Brazilian Health Regulatory Agency (ANVISA) guidelines and UPLC-Q-TRAP/MS [62] that followed the FDA bioanalytical method validation. In addition, the GC-FID method [60] had the lowest score in terms of validation criteria because the study lacks the full analytical data such as linearity, accuracy, precision and application in dosage forms of all drugs investigated in the study, actually, only EZE was considered which was measured in a linearity range of 39.98–999.60 μg/mL with strangely high LOD 19.992 μg/mL. Similar drawbacks were featured in the HPLC method [60]. Additional 20 merit points were assigned to the UPLC-Q-TRAP/MS [63] in the LOD and LOQ parameter for the analysis of unusual samples in the nano range as specified in the original RGB 12 algorithm study [3].

The green area exposed that the developed MEKC platform was the greenest with the minimum amount of reagents and waste production and lowest energy consumption followed by GC-FID [60], spectrophotometry [22], HPTLC [62], HPLC [60], UHPSFC [61] and finally UPLC-Q-TRAP/MS [63]. Noticeably, the aforementioned results elucidated the harmony with the AGREE assessment.

Regarding productivity and sustainability (blue area), the suggested MEKC was the topmost economic, fast and easily operated. Conversely, the reported UHPSFC [61] needed specific requirements for the use of supercritical fluid carbon dioxide. Moreover, UPLC-Q-TRAP/MS [63] had a multistep sample preparation since it was used for the determination of 13 hypolipidemic drugs in fingerprints, and GC-FID [60] comprised the manipulation of gases under pressure and high temperature. All the investigated methods employed automatic injectors, the measurements and applied procedures were presumed to be done in the same facility, and no transportation or special prerequisites were mentioned in the published studies.

To sum up, the proposed MEKC was the topmost with a total whiteness score of 96.3%. The collective results of both evaluation metrics revealed that the suggested MEKC technique was perfectly obeying the 12 principles of both GAC and WAC. Accordingly, it is considered a white, sustainable and ecofriendly platform for the analysis of the studied antihyperlipidemics.

## Conclusion

This is the first electro-driven separation method for multi-analyte concurrent analysis of these four antihyperlipidemic drugs. To our present knowledge, only very few reports describing HPLC–UV [60], GC-FID [60], UHPSFC [61] and UPLC-Q-TRAP/MS [63] methods fulfilled the task of simultaneous analysis of the 4 cited drugs. The proposed MEKC is a green and almost waste-free technique consuming low sample, solvent and energy. Thus, it is considered a fast, sensitive and greener alternative to other chromatographic platforms. Validation, greenness and sustainability are three pillars of pharmaceutical analysis that judge the usefulness of the analytical method. Furthermore, the ideal sustainable analytical method fulfilling the greenness criteria, analytical performance, cost and energy efficiency is recently called white. In these contexts, the implemented method is perfectly green and white analytical technique, obeying both GAC and WAC codes. It proved its excellent performance to be applied for rapid routine analysis of EZE in fixed-dose combination pills with ATO, ROS or SIM. Finally, this study is regarded one of the early applications of the comprehensive whiteness RGB 12 model in pharmaceutical analysis.

### Supplementary Information

**Additional file 1: Figure S1.** MEKC electropherogram of a standard mixture of ROS, ATO, EZE and SIM using 0.05M borate buffer pH 9.2. **Figure S2.** MEKC electropherogram of a standard mixture of ROS, ATO, EZE and SIM using 0.025M borate buffer pH 9.2 containing 0.025M SDS and 10%methanol. **Figure S3.** UV spectrum and purity plot for ROS. **Figure S4.** UV spectrum and purity plot for ATO. **Figure S5.** UV spectrum and purity plot for SIM. **Figure S6.** UV spectrum and purity plot for EZE. **Figure S7.** MEKC electropherogram of a sample solution obtained from Cholerose^®^ tablets containing 20 µg/mL ROS and 20 µg/mL EZE at 243 nm. **Figure S8.** MEKC electropherogram of a sample solution obtained from Atoreza^®^ tablets containing 40 µg/mL ATO and 40 µg/mL EZE at 243 nm. **Figure S9.** MEKC electropherogram of a sample solution obtained from Simv-Eze^®^ tablets containing 20 µg/mL SIM and 20 µg/mL EZE at 237 nm. **Figure S10.** Evaluation outcomes resulted from the RGB12 comparative study for the proposed MEKC method together with the published methods. The white bar indicates the arithmetic mean of the three other bars (red, green and blue). **Table S1.** Effect of buffer concentration on migration times of the four drugs. **Table S2.** System suitability parameters for MEKC-DAD analysis of ROS, ATO, EZE and SIM mixture. **Table S3.** Determination of ROS, ATO, SIM and EZE in laboratory-prepared mixtures using the proposed MEKC method.

## Data Availability

All data generated or analyzed during this study are included in this published article and its supplementary information file.
