# Supplementary material for: Development of a sustainable multianalyte MEKC method for quantitation of the antihyperlipidemic drugs ezetimibe together with three statins. Greenness and whiteness appraisal studies
Source: BMC Chem. 2023 Sep 23;17(1):124. doi: 10.1186/s13065-023-01040-y (PMC10518094; doi:10.1186/s13065-023-01040-y)
Supplement: Supplementary file 1 — Additional file 1: Figure S1. MEKC electropherogram of a standard mixture of ROS, ATO, EZE and SIM using 0.05M borate buffer pH 9.2. Figure S2. MEKC electropherogram of a standard mixture of ROS, ATO, EZE and SIM using 0.025M borate buffer pH 9.2 containing 0.025M SDS and 10%methanol. Figure S3. UV spectrum and purity plot for ROS. Figure S4. UV spectrum and purity plot for ATO. Figure S5. UV spectrum and purity plot for SIM. Figure S6. UV spectrum and purity plot for EZE. Figure S7. MEKC electropherogram of a sample solution obtained from Cholerose® tablets containing 20 µg/mL ROS and 20 µg/mL EZE at 243 nm. Figure S8. MEKC electropherogram of a sample solution obtained from Atoreza® tablets containing 40 µg/mL ATO and 40 µg/mL EZE at 243 nm. Figure S9. MEKC electropherogram of a sample solution obtained from Simv-Eze® tablets containing 20 µg/mL SIM and 20 µg/mL EZE at 237 nm. Figure S10. Evaluation outcomes resulted from the RGB12 comparative study for the proposed MEKC method together with the published methods. The white bar indicates the arithmetic mean of the three other bars (red, green and blue). Table S1. Effect of buffer concentration on migration times of the four drugs. Table S2. System suitability parameters for MEKC-DAD analysis of ROS, ATO, EZE and SIM mixture. Table S3. Determination of ROS, ATO, SIM and EZE in laboratory-prepared mixtures using the proposed MEKC method. [file 13065_2023_1040_MOESM1_ESM.pdf]

Development of a sustainable multianalyte MEKC method for quantitation of the antihyperlipidemic drugs ezetimibe together with three statins. Greenness and whiteness appraisal studies

Additional file materials

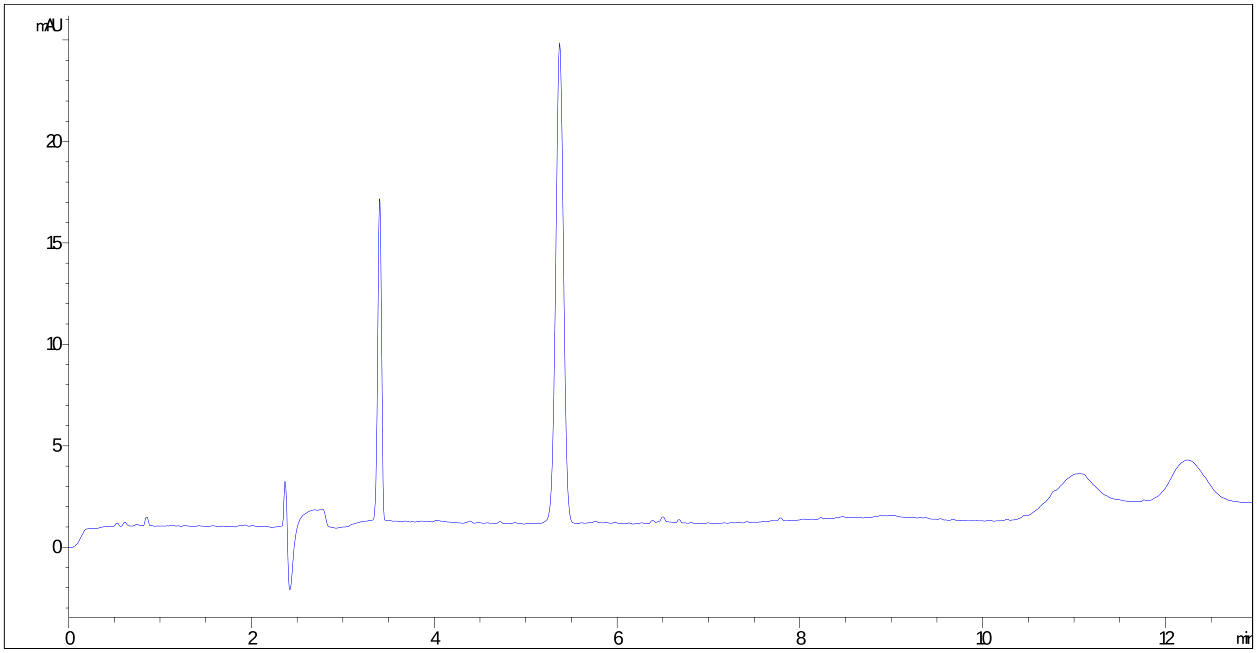

Figure 1S: MEKC electropherogram of a standard mixture of ROS, ATO, EZE and SIM using 0.05M borate buffer pH 9.2.

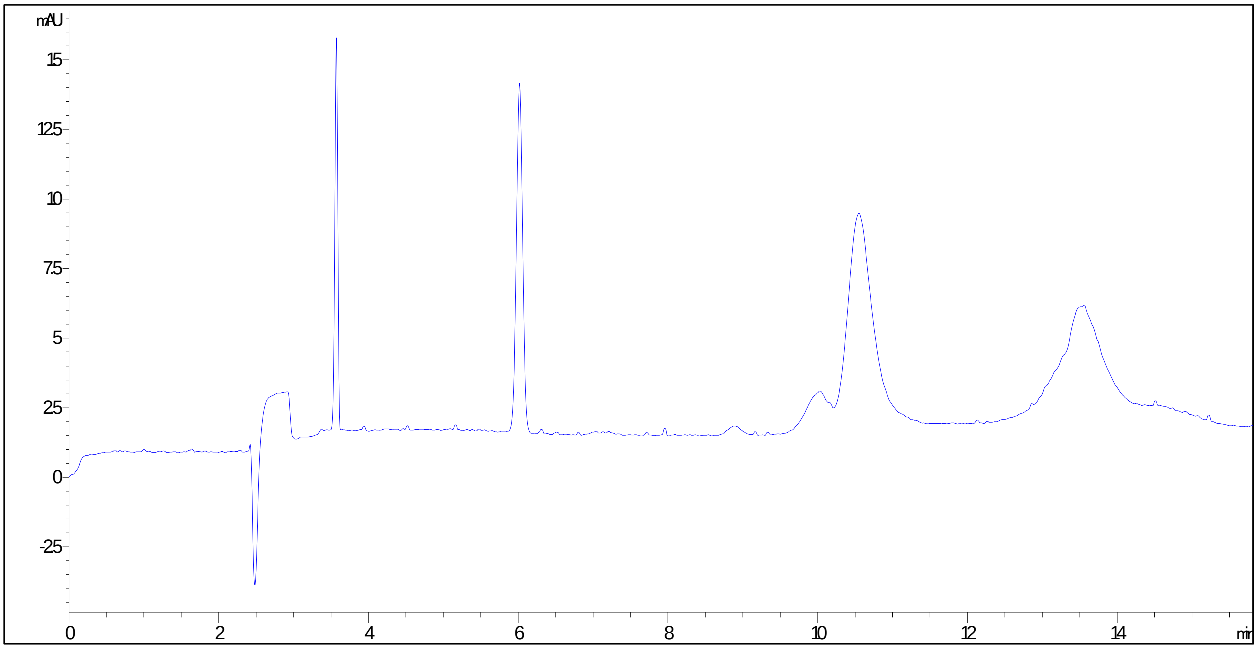

Figure 2S: MEKC electropherogram of a standard mixture of ROS, ATO, EZE and SIM using 0.025M borate buffer pH 9.2 containing 0.025M SDS and 10%methanol.

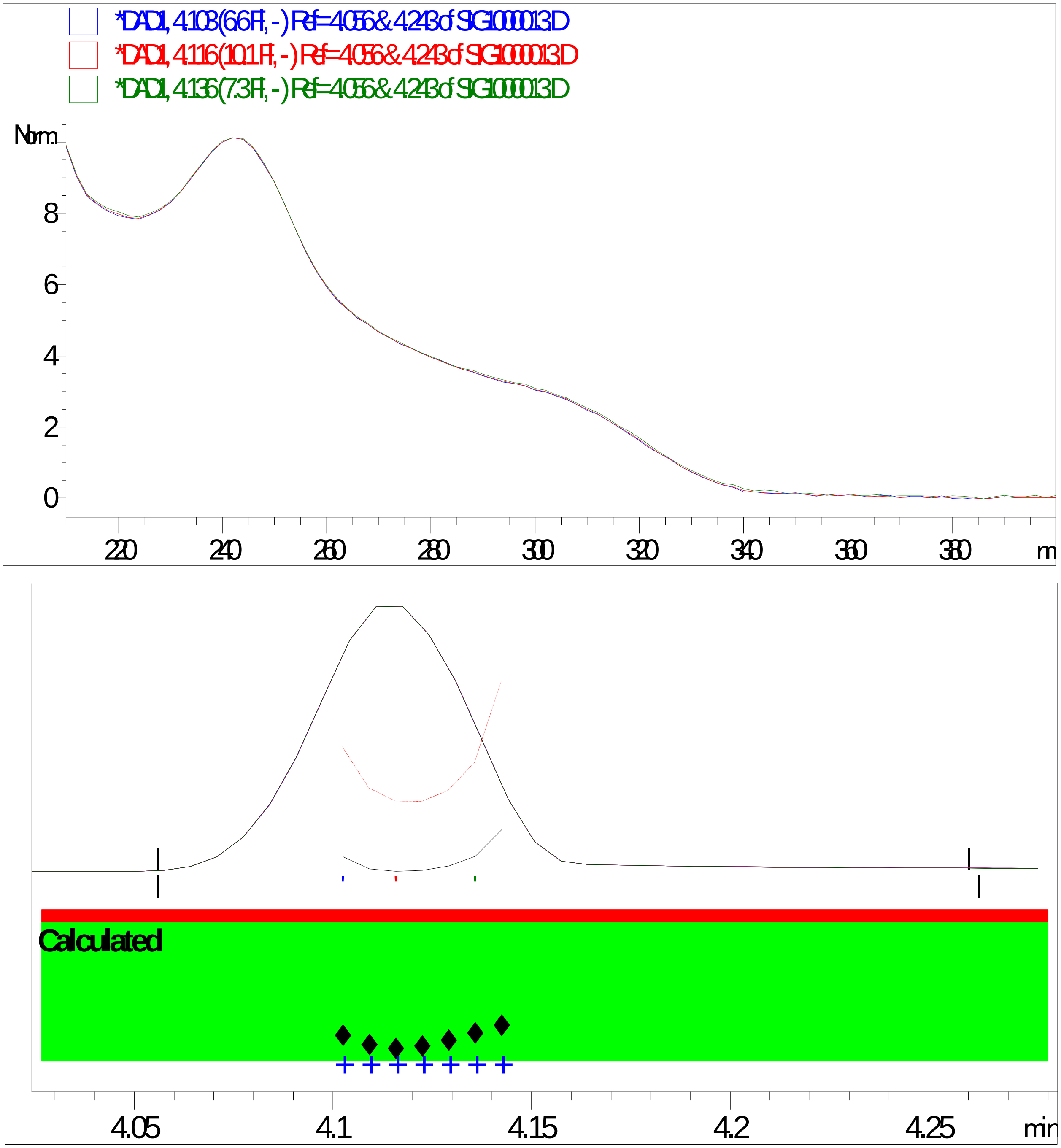

Figure 3S: UV spectrum and purity plot for ROS

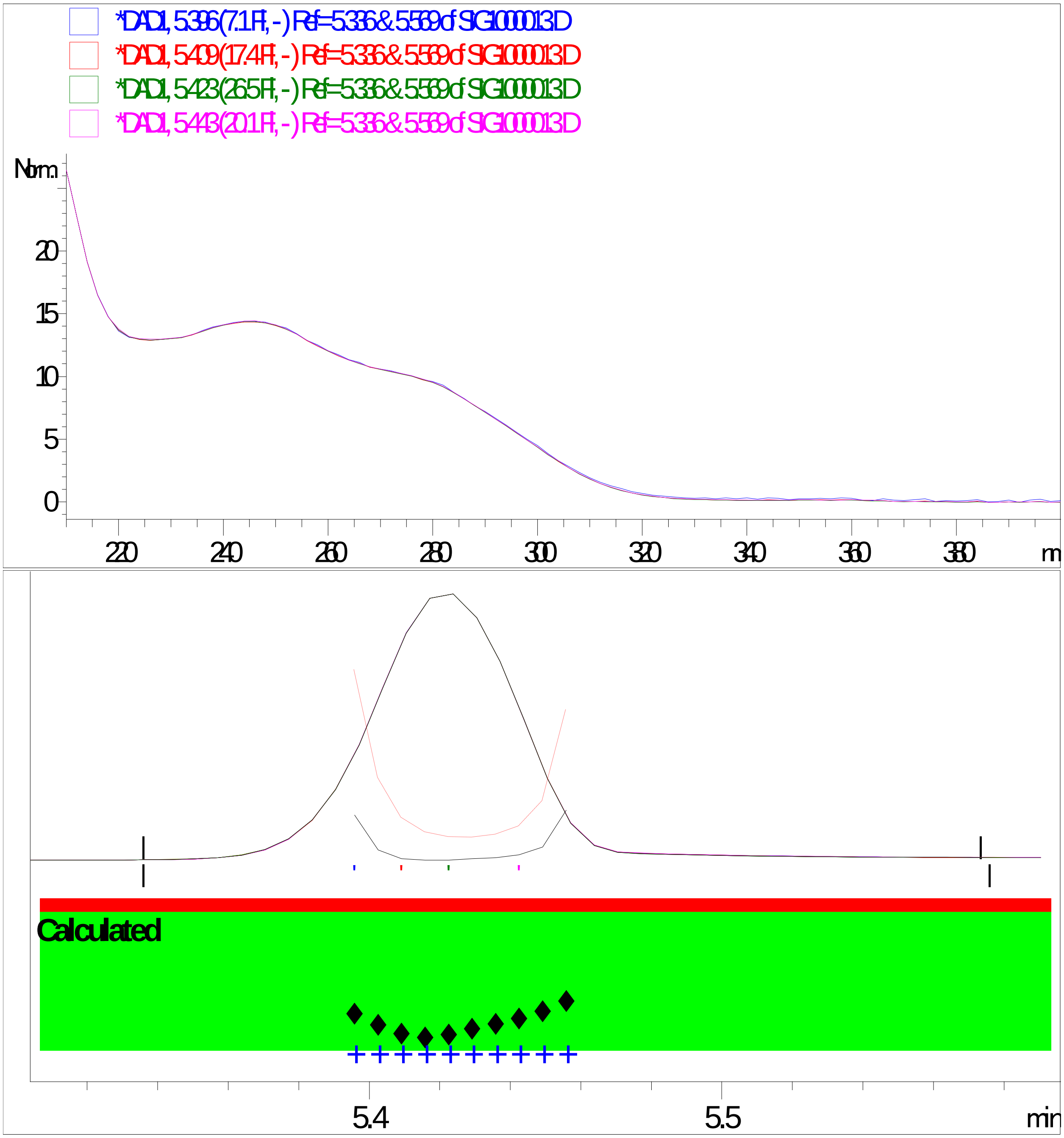

Figure 4S: UV spectrum and purity plot for ATO

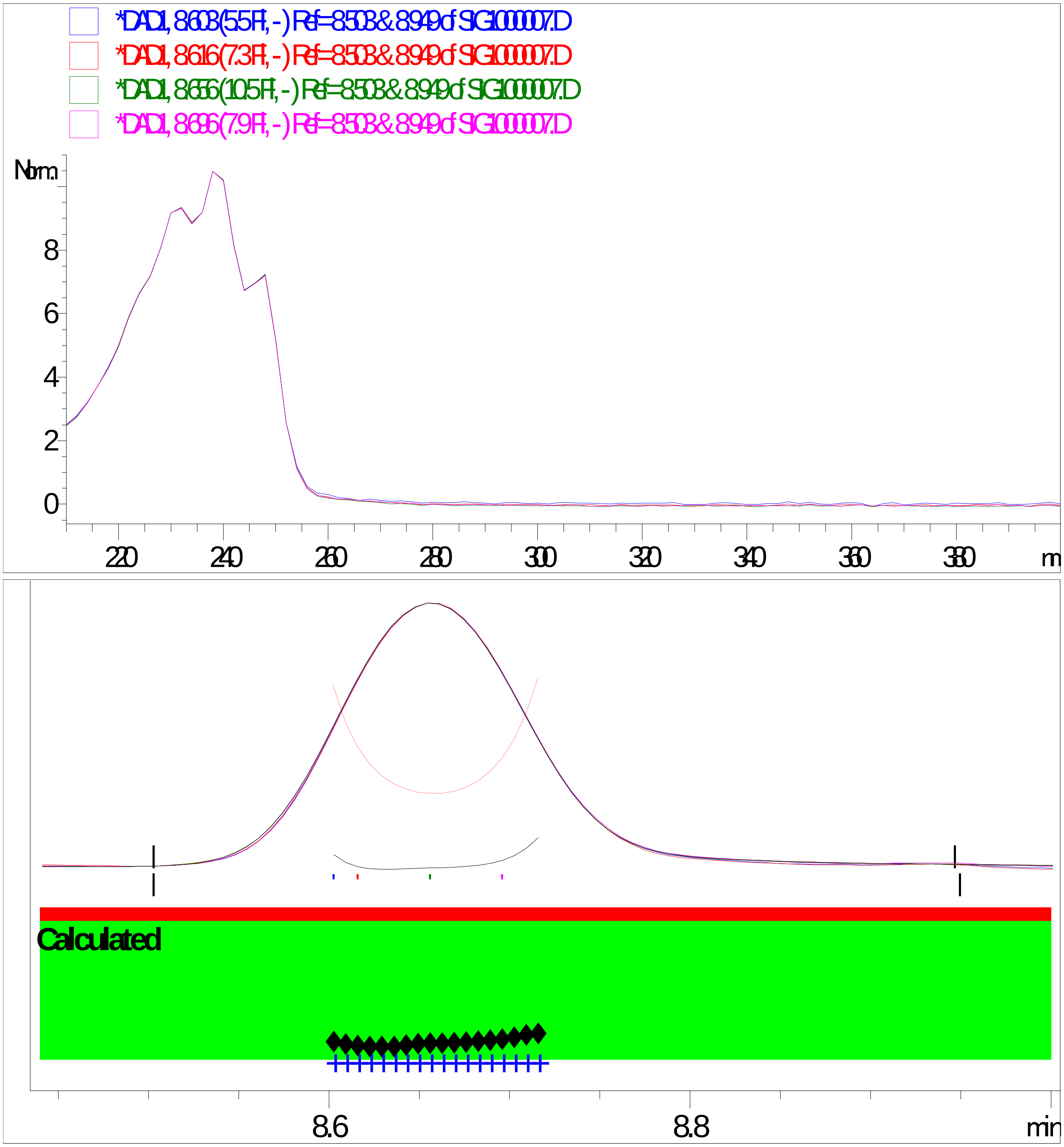

Figure 5S: UV spectrum and purity plot for SIM

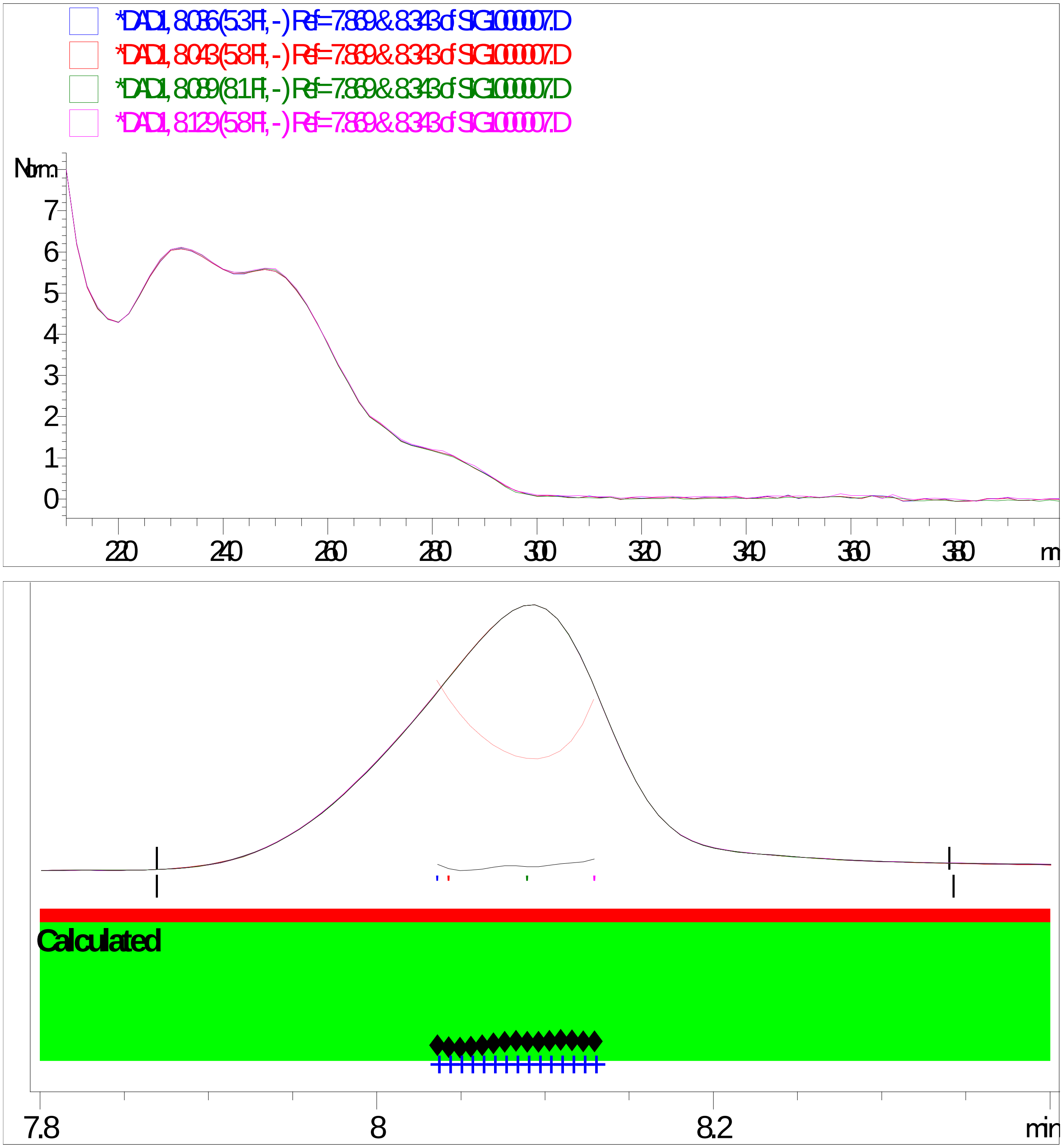

Figure 6S: UV spectrum and purity plot for EZE

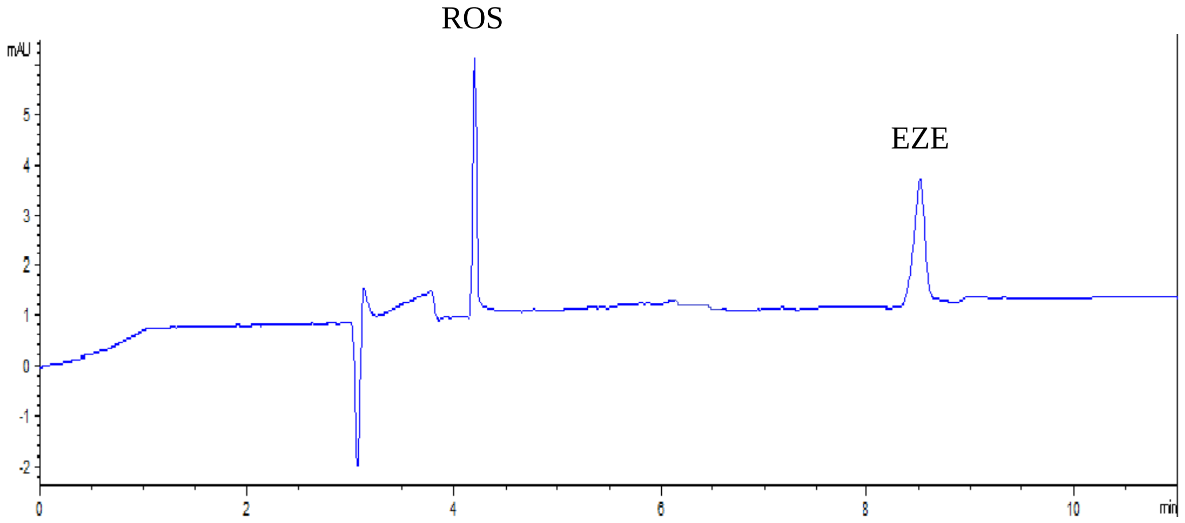

Figure 7S: MEKC electropherogram of a sample solution obtained from Cholerose® tablets containing 20 µg/mL ROS and 20 µg/mL EZE at 243 nm.

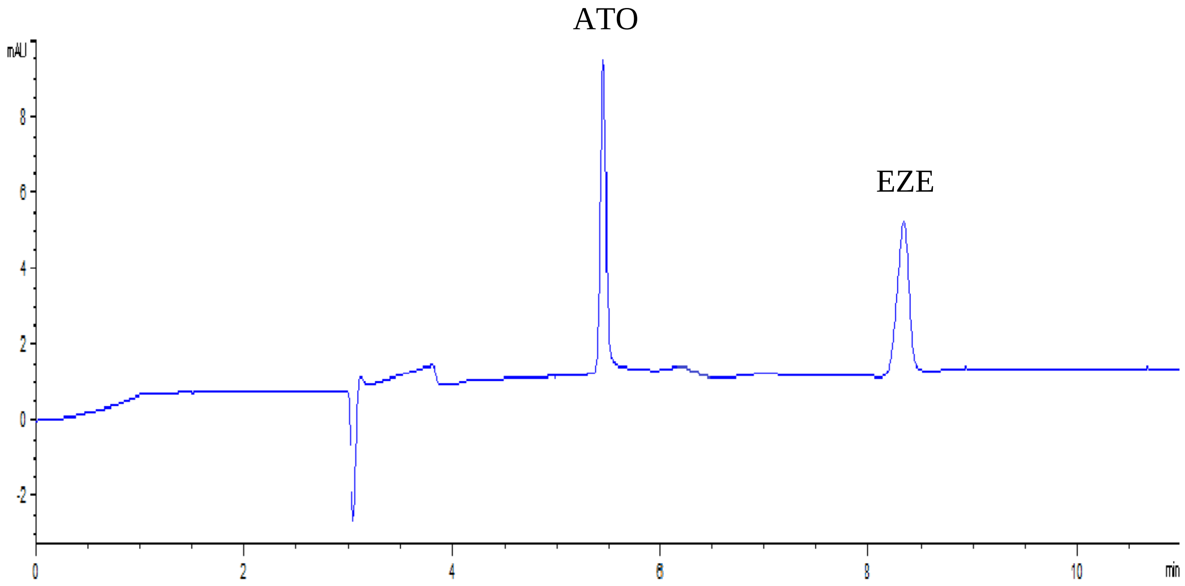

Figure 8S: MEKC electropherogram of a sample solution obtained from Atoreza® tablets containing 40 µg/mL ATO and 40 µg/mL EZE at 243 nm.

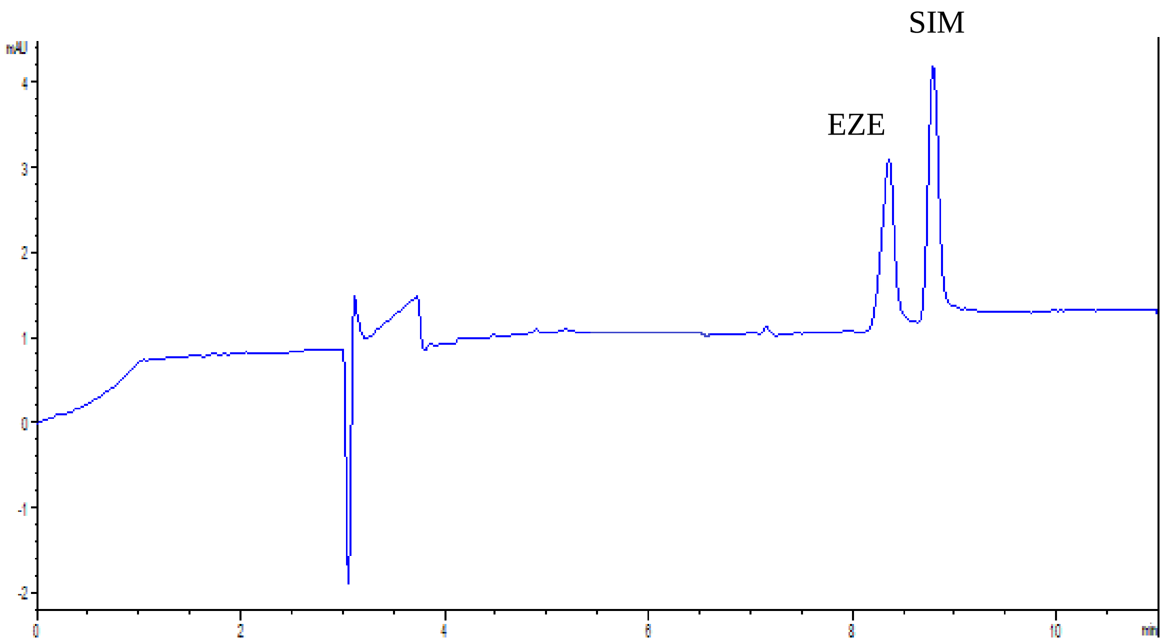

Figure 9S: MEKC electropherogram of a sample solution obtained from Simv-Eze® tablets containing 20 µg/mL SIM and 20 µg/mL EZE at 237 nm.

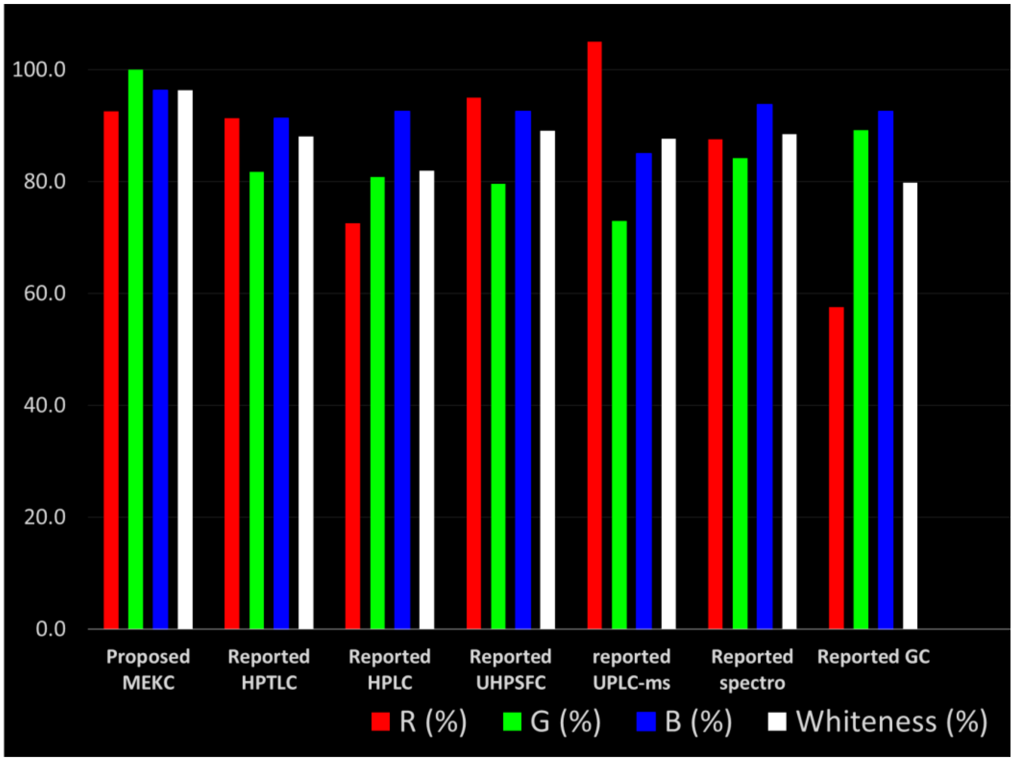

Figure 10S: Evaluation outcomes resulted from the RGB12 comparative study for the proposed MEKC method together with the published methods. The white bar indicates the arithmetic mean of the three other bars (red, green and blue).

Table 1S: Effect of buffer concentration on migration times of the four drugs

Concentration of buffer (M)	Migration time (min)			
	ROS	ATO	EZE	SIM
**0.010**	2.96	4.91	6.13	6.34
**0.025**	4.12	5.42	8.23	8.74
**0.050**	3.51	5.65	11.1	12.31

Table 2S: System suitability parameters for MEKC-DAD analysis of ROS, ATO, EZE and SIM mixture

Parameter	ROS	ATO	EZE	SIM
**Migration time ± SD (min)**	4.12 ± 0.04	5.42 ± 0.014	8.23 ± 0.06	8.74 ± 0.04
**Retention factors (k')**	3.09	4.41	7.16	7.51
**Theoretical plates (N)**	43181	70810	29238	35343
**USP tailing factor**	0.91	0.96	1.34	0.93
**Selectivity (α)**	–	1.32	1.52	1.06
**Resolution (R_s)**	–	16.23	20.45	2.64

Table 3S: Determination of ROS, ATO, SIM and EZE in laboratory-prepared mixtures using the proposed MEKC method

Nominal value (µg/ml)				Found ± SD[a] (µg/ml)				RSD(%)[b]				E_r(%)[c]			
ROS	ATO	EZE	SIM	ROS	ATO	EZE	SIM	ROS	ATO	EZE	SIM	ROS	ATO	EZE	SIM
20	20	80	80	20.01 ±0.14	19.97 ±0.32	80.08 ±0.51	80.21 ±0.09	0.70	1.60	0.64	0.11	0.05	-0.15	0.10	0.26
40	40	60	60	40.02 ±0.17	39.86 ±0.42	60.33 ±0.16	59.92 ±0.16	0.43	1.05	0.27	0.27	0.05	-0.35	0.55	-0.13
60	60	40	40	60.18 ±0.30	60.35 ±0.15	39.89 ±0.38	40.31 ±0.24	0.50	0.25	0.95	0.60	0.30	0.58	-0.27	0.78
80	80	20	20	80.44 ±0.46	80.08 ±0.25	20.35 ±0.16	20.05 ±0.19	0.57	0.31	0.79	0.95	0.55	0.10	1.75	0.25
20	20	20	20	20.17 ±0.38	20.06 ±0.08	20.31 ±0.19	20.14 ±0.14	1.88	0.40	0.94	0.70	0.85	0.30	1.55	0.70

[a] Mean ± standard deviation for three determinations.
[b] % Relative standard deviation.
[c] % Relative error.
